# Identification of a novel MAGT1 mutation supports a diagnosis of XMEN disease

**DOI:** 10.1038/s41435-022-00166-8

**Published:** 2022-03-09

**Authors:** Christopher M. Watson, Fatima Nadat, Sammiya Ahmed, Laura A. Crinnion, Sean O’Riordan, Clive Carter, Sinisa Savic

**Affiliations:** 1 North East and Yorkshire Genomic Laboratory Hub, The Leeds Teaching Hospitals NHS Trust, St. James’s University Hospital, Beckett Street, Leeds, LS9 7TF UK; 2 Leeds Institute of Medical Research, University of Leeds, St. James’s University Hospital, Beckett Street, Leeds, LS9 7TF UK; 3 Department of Clinical Immunology and Allergy, St James’s University Hospital, Beckett Street, Leeds, LS9 7TF UK; 4 Department of Paediatric Immunology, Leeds General Infirmary, Leeds, LS1 3EX UK; 5 National Institute for Health Research, Leeds Biomedical Research Centre and Leeds Institute of Rheumatic and Musculoskeletal Medicine (LIRMM), Wellcome Trust Brenner Building, St James’s University Hospital, Beckett Street, Leeds, LS9 7TF UK

**Keywords:** Immunology, Primary immunodeficiency disorders

## Abstract

XMEN (X-linked immunodeficiency with magnesium defect) is caused by loss-of-function mutations in *MAGT1* which is encoded on the X chromosome. The disorder is characterised by CD4 lymphopenia, severe chronic viral infections and defective T-lymphocyte activation. XMEN patients are susceptible to Epstein-Barr virus infections and persistently low levels of intracellular Mg^2+^. Here we describe a patient that presented with multiple recurrent infections and a subsequent diffuse B-cell lymphoma. Molecular genetic analysis by exome sequencing identified a novel hemizygous *MAGT1* nonsense mutation c.1005T>A (NM_032121.5) p.(Cys335*), confirming a diagnosis of XMEN deficiency. Follow-up immunophenotyping was performed by antibody staining and flow cytometry; proliferation was determined by ^3^H-thymidine uptake after activation by PHA and anti-CD3. Cytotoxic natural killer cell activity was assessed with K562 target cells using the NKTEST^TM^ assay. While lymphocyte populations were superficially intact, B cells were largely naive with a reduced memory cell compartment. Translated NKG2D was absent on both NK and T cells in the proband, and normally expressed in the carrier mother. In vitro NK cell activity was intact in both the proband and his mother. This report adds to the growing number of identified XMEN cases, raising awareness of a, still rare, X-linked immunodeficiency.

## Introduction

X-linked immunodeficiency with magnesium defect (XMEN; OMIM: 300853) is a complex primary immunodeficiency (PID) caused by pathogenic loss-of-function variants in *MAGT1* [1]. The clinical manifestation of XMEN is variable, with patients typically characterised by an increased susceptibility to Epstein-Barr virus (EBV), a high viral load, and subsequent EBV-associated lymphomas. Patients also experience increased susceptibility to sinopulmonary and ear infections, although these are typically mild. The immunological phenotype includes an increased CD8^+^ T-cell count (with an associated CD4:CD8 inversion), and an elevated B-cell count. T-cell function defects have also been observed; these include absence of the natural killer stimulatory receptor, NKG2D, on both natural killer (NK) and CD8^+^ T cells [2].

Magnesium transporter 1 (MAGT1) is an endoplasmic reticulum resident Mg^2+^ transporter, important for ensuring homoeostasis of intracellular magnesium [1]. Early studies demonstrated that loss of MAGT1 results in a decrease in free intracellular Mg^2+^ [3]. More recently, MAGT1 has been identified as a non-catalytic accessory protein, crucial for the function of the oligosaccharide transferase (OST) complex, which is itself responsible for asparagine (N)-linked glycosylation [4]. Either MAGT1 or its homologue TUSC3, associate with the enzymatic subunit of the OST complex, STT3B, prior to the post-transitional transfer of glycans to the N-linked glycosylation site. Since immune cells do not express TUSC3, N-linked glycosylation of STT3B-dependent glycoproteins are reliant on MAGT1. Mutations in MAGT1 therefore manifest as an immunological disorder, despite the functional effect being primarily associated with defective glycosylation. This was recently demonstrated in a cohort of 23 XMEN-confirmed patients [5]. A consequence of reduced glycosylation was the absence of the NK cell receptor, NKG2D (on both NK cells and T cells). This receptor plays a crucial role in NK cell activation following EBV infection [6].

In contrast to many T-cell immunodeficiencies, XMEN does not present with severe life-threatening infections. Instead, mortality of XMEN disease is linked to chronic EBV-associated malignancies, which may not develop until the second decade of life. A high index of suspicion is therefore necessary to make a clinical diagnosis of primary immunodeficiency. Here we report how genetic analysis identified a novel pathogenic *MAGT1* mutation, causing XMEN deficiency in a child who had long evaded diagnosis.

## Materials and methods

DNA was isolated from peripheral blood lymphocytes using a bead-based extraction method, following written consent. Ethical approval for this study was granted by the Leeds East Research Ethics Committee (18/YH/0070). Whole-exome sequencing using SureSelect v6 hybridisation capture baits (Agilent Technologies, Wokingham, UK) combined with paired-end 151-bp short-reads generated using sequencing-by-synthesis chemistry (Illumina, Inc., CA USA). Data processing was performed using a standard informatics workflow, as previously described [7]. Sanger sequencing primers and thermocycling conditions used to validate next-generation sequencing findings are available upon request.

Lymphocyte phenotyping, using our standard clinical panel, was performed to determine the percentage and absolute number of CD3^+^, CD4^+^, and CD8^+^ T cells, CD19^+^ B cells, and CD3^−^ CD56^+^ NK cells. In brief, whole blood was added to flourochrome labelled antibodies that recognised cell surface determinants on T, B and NK cells (CD45/CD3/CD4/CD8/CD19/CD16-56) (Becton Dickinson, Berkshire, UK). Following erythrocyte lysis, the samples were acquired on a FACSCanto^TM^ II flow cytometer using BD FACSCanto^TM^ Clinical Software v.3 (Becton Dickinson). TruCount^TM^ tubes (Becton Dickinson) were used to determine the percentage and absolute value of the subsets measured.

B cells were further analysed using antibody staining and flow cytometry to assess naive (CD27^−^IgD^+^) and memory (CD27^+^IgD^+/−^) phenotypes in addition to CD24^hi^CD38^hi^ transitional B-cell populations. Cell surface markers (CD3^+^CD4^+^CD45RA^+^CD27^+^) were used to assess naive T cells and the T-cell surface HLA-DR expression was analysed. Additionally, the percentage of naive T cells considered to be recent thymic emigrants (CD3^+^CD4^+^ CD45RA^+^CD31^hi^) were identified. NKG2D expression was assessed on CD3^-^CD56^+^ NK cells, CD3^+^ T cells and CD3^+^CD56^+^ NKT cells. For all phenotyping, whole blood was added to antibody mixes (outlined above) for 20 min in the dark at room temperature (for B-cell analysis blood was washed twice in 2.5 mL of PBS + 1% FBS by centrifugation at 330 × *g* for 6 min prior to staining with antibodies). Following red cell lysis, the samples were washed twice (as described), after which the cell pellet was resuspended in approximately 300 µL phosphate-buffered saline + 0.5% formaldehyde. Results were acquired using a FACSCanto^TM^ II flow cytometer with BD FACSDiva^TM^ v.8 software (Becton Dickinson). Specific subset analysis (as described above) was carried out on the lymphocyte population as determined by FSC/SSC.

To assess T-cell proliferation, peripheral blood mononuclear cells (PBMCs) were separated from whole heparinised blood using Lympholyte^®^-H (Cedarlane, NC, USA), prior to seeding, into 96-well plates, at a density of 1 × 10^5^ cells/well, containing increasing concentrations of phytohemaglutinin (Sigma-Aldrich, MO, USA). For anti-CD3 stimulation, cells were plated into anti-CD3 coated wells (Scientific Laboratory Supplies Ltd., UK). Following culture, ^3^H-thymidine (Perkin Elmer, MA, USA) was added to the cells 16 h prior to harvest, which was performed using a Scatron AS semi-automated cell harvester. Incorporated ^3^H activity was assessed from dried filters placed in *Optiphase Hisafe 3* scintillant (Perkin-Elmer, UK) using a B counter (Wallac LKB B counter). Results were expressed as incorporated counts minus background (unstimulated cells), with the mean of three replicates being averaged for each experimental condition.

NK cell activity against K562 target cells was evaluated using the NKTEST^TM^ assay (Glycotope Biotechnology GmbH). PBMCs were isolated from heparinised blood and resuspended to a density of 5 × 10^6^ cells/tubein 5 mL polystyrene round bottom tubes. Fluorescently labelled K562 cells (10^5^/mL) were added to the isolated PBMCs to create a decreasing ratio of PBMC:K562 cells (50:1, 25:1 and 12.5:1, respectively). The mixed cell suspensions were incubated in a water bath for 4 h at 37 °C, 5% CO_2_. Cells were subsequently stained using a DNA dye, which enters only those cells with a compromised plasma membranes, allowing the separation of killed K562 cells by flow cytometry. Results were acquired using a FACSCanto^TM^ II flow cytometer with BD FACSDiva^TM^ v.8 software. Percentage specific cytotoxicity was calculated as the proportion of dead K562 cells, relative to a negative control.

## Results

A paediatric male patient was referred to the immunology clinic for investigations of recurrent chest infections that required multiple courses of intravenous antibiotics since the age of 4; on one occasion this included culture proven streptococcal sepsis. It was noted that from a young age he had ongoing issues with widespread disseminated scarring molluscum contagiosum, with more than 100 lesions at its peak.

His mother reported that he had also previously suffered from a prolonged outbreak of varicella-zoster virus, with new lesions forming over a 2-week period (but this did not require hospitalisation). He had received all vaccines (including live vaccines) with no adverse effects. There were no concerns regarding failure to thrive, and he had reached all other milestones as expected with no intellectual delays. His respiratory symptoms had improved significantly with age.

He suffered chronically loose stools, opening his bowels up to 3 times a day; with no impact on growth (indeed BMI has rapidly increased with weight on 99.6%). Investigations for infectious and inflammatory causes has been negative, together with a coeliac screen.

At the age of 6, his tetanus and pneumococcal antibody titres were low. These responded to booster vaccinations but antibody levels were not maintained. His Haemophilus B antibody levels have always been within adequate range.

A follow-up review identified a palpable right cervical lymph node, with concerning features, which was confirmed to be diffuse B-cell lymphoma. He was positive for EBV at diagnosis, and after completion of a chemotherapy regime, which included rituximab, he has had a persistently high viral load (7000–18,500 IU/mL).

At the age of 9, he underwent three cycles of chemotherapy, which were complicated by significant neutropenic sepsis; culprit organisms included human metapneumovirus and parainfluenza type 1, requiring critical care admission on two occasions for respiratory support. Post chemotherapy repeat imaging confirmed that he had complete resolution of his lymphoma, with a good radiological and clinical response. Chest computed tomography scans, undertaken for oncological assessment, revealed no bronchiectasis.

**Fig. 1:**
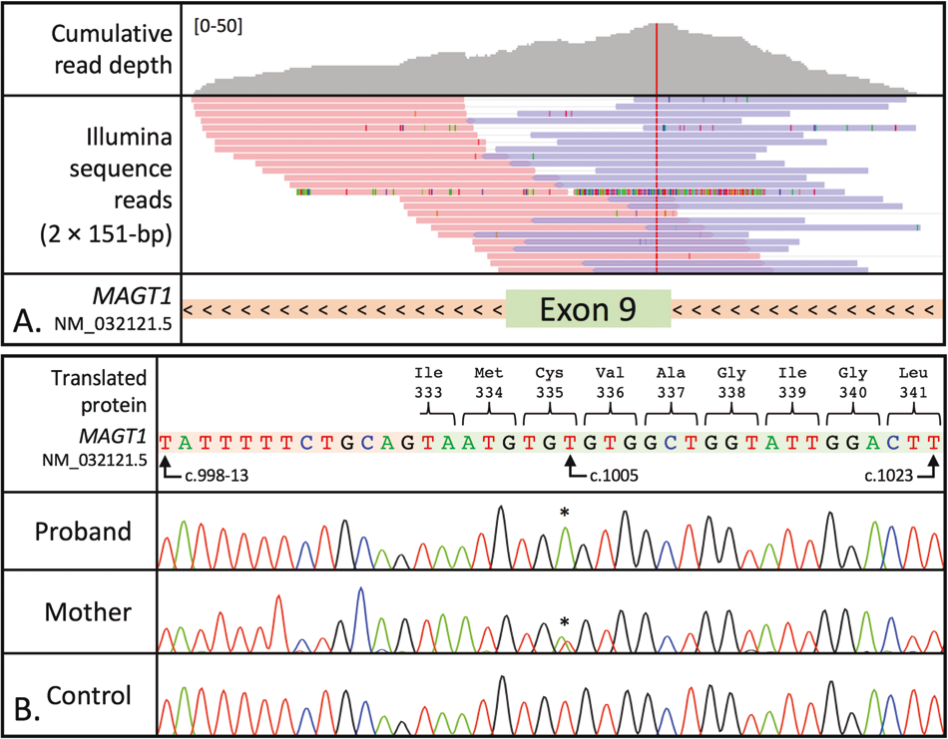
Overview of genetic investigations. **A** Next-generation sequencing reads supporting the identification of the hemizygous variant c.1005T>A p.(Cys335*) in *MAGT1* exon 9 (NM_032121.5). The cumulative read count is displayed per nucleotide (the *y*-axis scale is 0–50). Read pairs are aligned to the human reference sequence (hg19) and are coloured by read strand; pink denotes a positive rightward (5′-3′) DNA strand and blue denotes a negative leftward (reverse complement) DNA strand. Non-reference (mismatched) bases are highlighted within each alignment. *MAGT1* is encoded on the antisense strand; left-facing arrows (<) indicate the direction of transcription. **B** Sanger sequencing chromatograms showing the hemizygous *MAGT1* variant c.1005T>A p.(Cys335*) identified in the proband and his carrier mother. Variant nomenclature is reported according to reference transcript NM_032121.5. * denotes the variant nucleotide.

At 13 years of age, the patient was reviewed for molecular genetic diagnosis of a suspected primary immunodeficiency (indicative clinical features included high EBV titres, lymph-proliferative disease, and recurrent severe infections). The patient is of non-consanguineous white British ancestry and there was no notable family history (although the father died in his 40 s following a diagnosis of pneumonia). The sequencing library yielded 18,040,641 read pairs, of which 5.17% were classified as PCR duplicates. The 33,457 identified DNA sequence variants were triaged using a comprehensive 233-gene PID virtual panel (curated using PanelApp, a knowledge base of disease-associated genes [8]). This decreased the number of identified variants to 475. Standard filtering criteria further excluded variants that were predicted to cause a synonymous amino acid change, had a reported gnomAD [9] allele frequency ≥1% or were located in upstream, intronic or untranslated regions, reducing the analytical burden to 10 candidate variants. Clinical interpretation, in accordance with ACMG guidelines [10], supported disease-associated pathogenicity for the hemizygous variant, c.1005T>A (NM_032121.5) p.(Cys335*), in *MAGT1* exon 9 (Fig. 1A). The X-chromosome variant, creates a premature stop codon, that is predicted to cause premature termination of the protein. In addition to being absent from both in-house and publicly available variant databases, the variant represents a novel mutation that has not been previously described to cause XMEN deficiency. Sanger sequencing validated the NGS result and provided confirmation that the mutation was maternally inherited (Fig. 1B). From a clinical management perspective, we note that fewer than 25 patients have, to-date, been reported with molecularly confirmed XMEN deficiency [11]; their clinical and immunological characteristics are summarised in Table 1.

**Table 1:** Clinical and immunological features of reported XMEN cases.

Reference	Age at diagnosis	Infections	Malignancy	EBV	CD4:CD8 ratio	CD4 (%/counts)	B cells (%)	IgG mg/dL	IgA mg/dL	IgM mg/dL	Other features	Outcome
[3]	7 years	EBV, Herpes simplex virus, Viral pneumonia, Otitis media, Sinusitis	None	+	0.6 ↓	13.5 ↓	37.1 ↑	1160 N	87 N	92 N	Splenomegaly, Diarrhoea, Automimmune cytopenia	Alive
	3 years	EBV, Herpes simplex virus, Viral pneumonia, Otitis media, Sinusitis	None	+	0.7 ↓	27.8 ↓	20.8 N	286 ↓	7 ↓	55 N	Splenomegaly, Diarrhoea	Alive
	45 years	EBV	Unspecified lymphoma	+	8.5 ↑	74.4 ↑	14.1 N	734 N	128 N	14 ↓	Splenomegaly, Chronic active EBV hepatitis, Pancytopenia, Haemophagocytosis	Deceased (HSCT)
	4 years	EBV, Sinusitis	None	+	0.6 ↓	U	U	1030 N	56 N	115 N	Splenomegaly	Alive
	16 years	EBV, Otitis media, Molluscum contagiosum	Burkitt’s lymphoma (x2)	+	0.5 ↓	17 ↓	0↓(RTX)	611 ↓	35.6 ↓	87 N	Splenomegaly	Alive
	16 years	EBV, Strep pharyngitis, Epiglottitis	B-cell lymphoproliferative disease	+	0.55 ↓	19.1 ↓	46 ↑	1690 ↑	14.8 ↓	29 ↓	Splenomegaly, Autoimmune cytopenia	Alive
	23 years	EBV, Otitis media, Streptococcal pharyngitis, Varicella, Recurrent zoster, Pertussis	Hodgkin lymphoma (x2)	+	1.1 N	40 N	44 ↑	619 ↓	29.9 ↓	38 ↓	Splenomegaly	Deceased (HSCT)
[22]	36 years	Recurrent respiratory infections, Bronchiectasis	B-cell lymphoproliferative disease	+	0.76 ↓	0.95 N	U	900 N	121 N	126 N	Hepatosplenomegaly, Progressive multifocal leucoencephalopathy post chemotherapy/rituximab, Thrombocytopenia	Deceased
	13 years	No history of recurrent infections	B-cell lymphoproliferative disease	+	0.87 ↓	0.62 N	U	462 ↓	33 ↓	44 N		Alive
[23]	17 years	Recurrent sinopulmonary infections, CMV infection	Hodgkin lymphoma	+	↓	↓	U	880 N	50 N	52 N	Guillan-Barré syndrome, Idiopathic thrombocytopenic prupura, Autoimmune haemolytic anaemia, Autoimmune hepatitis	Deceased (HSCT)
[24]	6 years	Recurrent sinopulmonary infections, Bronchiectasis, HHV8	Kaposi’s sarcoma	+	U	↓	↓	↓	↓	↓	Gallstones	Alive, Remission
[25]	13 years	No history of recurrent infections	None	–	U	N	U	U	U	U	Intellectual/developmental delay, Mild facial dysmorphism, Behaviour abnormalities	Alive
	11 years	No history of recurrent infections	None	–	U	U	U	U	U	U	Intellectual/developmental delay, Mild facial dysmorphism, Hepatomegaly	Alive
	17 years	EBV, Other non-specified infections	None	+	N	↓	U	U	U	U		Alive
[26]	29 years	Recurrent sinopulmonary infections, Bronchiectasis	B-cell lymphoproliferative disease, Liposarcoma	+	U	U	U	↓	U	U	ITP, Major epistaxis, upper GI bleed, SAH and significant other bleeding history, Seizures, BK virus haemorrhagic cystitis post BMT aplasia	Alive (HSCT)
	17 years	No history of recurrent infections	B-cell lymphoproliferative disease	+	U	U	U	U	U	U	Frequent epistaxis, BK virus - haemorrhagic cystitis post BMT aplasia	Alive (HSCT)
	20 years	No history of recurrent infections	B-cell lymphoproliferative disease	+	U	U	U	↓	U	U	AIHA, epistaxis, Steatohepatitis, Panhypopituitarism secondary to EBV LPD, Obesity, BK virus - haemorrhagic cystitis post BMT aplasia	Deceased (HSCT)
[27]	15 years	Herpes zoster, CMV, BK virus, Pansinusitus	Hodgkin lymphoma	+	1.6 N	1.0 N	N	585 ↓ (IVIG)	24 ↓	77 N	Epistaxis, Splenomegaly, Immune thrombocytopenic purpura	Alive, Remission
	4 months	Pneumocystis jirovecii, CMV	None	-	1.2 N	1.0 ↓	N	811 ↑	17 ↓	127 ↑		Alive, Remission
[14]	31 years	Severe meningoecephalitis, Recurrent sinopulmonary infections, Varicella	Hodgkin lymphoma (x2), Burkitt’s lymphoma, Atypical lymphoproliferative cutaneous lesion	+	0.62 ↓	↓	↑	140 ↓	119 N	324 N		Alive
	12 years	Recurrent sinopulmonary infections, Chronic bronchitis, Disseminated molluscum contagiosum	Castleman disease	–	0.87 ↓	↓	↑	530 ↓	18 ↓	90 N	Middle cerebral artery vasculitis, Eosinophilic esophagitis	Alive (HSCT)
Reported patient	13 years	Recurrent sinopulmonary infections, Molluscum contagiousum, Significant history of varicella	B-cell lymphoma	+	0.82 ↓	N	N	N	N	N	Obesity, Chronic diarrhoea	Alive

↓: Below normal range. ↑: Above normal range.

*EBV* Epstein-Barr virus, *HSCT* haematopoetic stem cell transplant, *N* normal, *U* unknown, *RTX* rituximab, *IVIG* intravenous immunoglobulin, *N* Normal.

Lymphocyte subset analysis was performed over a 6-year period (2015–2021) to determine the percentage and absolute numbers of T, B, and NK cells. While T-cell numbers were within age-adjusted ranges, at all timepoints tested, an expansion of CD8 T cells generated the typical CD4:CD8 inversion associated with XMEN disease (Supplementary Fig. 1A). The patient had normal naive (CD3^+^CD4^+^CD45RA^+^CD27^+^) T-cell numbers (79.6% in 2021) and over 95% of T cells were HLA-DR negative. Double negative (CD3^+^CD4^−^CD8^−^) TCRγδ^+^ T cells were found to be within the normal range (4.8% in 2021) and explained any discrepancy in the CD3/4/8 T-cell compartment observed in the lymphocyte cell marker assay. Proliferation in response to the T-cell mitogen PHA and anti-CD3 was assessed by ^3^H-thymidine uptake; this was consistent with a normal profile (three replicates performed), Supplementary Fig. 1B.

While B-cell numbers were broadly consistent with age-adjusted reference values, a large proportion of B lymphocytes (94.4%) were CD27^-^ naive, as has been previously described [5]. We further observed that there was a lower than expected proportion of CD27^+^IgD^+^ non-switched and CD27^−^IgD^−^ switched memory B cells (2.4% and 1.4%, respectively, Supplementary Fig. 2B). It is notable that in addition to a poor vaccine response to Tetanus, Hemophilus and Pneumococcus, low immunoglobulins levels were measured (Supplementary Fig. 2C). Finally, an elevated percentage of transitional B cells (CD24^hi^CD38^hi^) was observed (February: 12.9%; June: 11.5%, Sept: 13.9%).

**Fig. 2:**
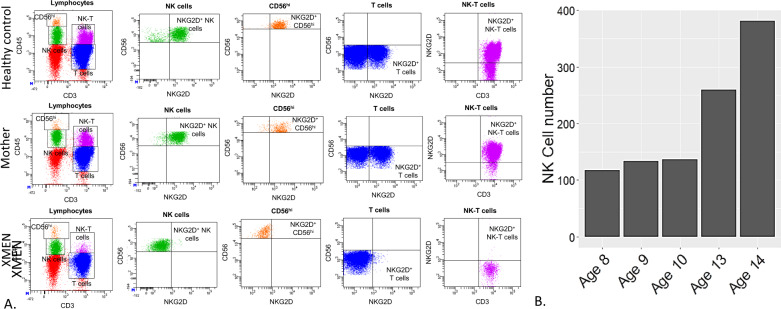
Lymphocyte phenotyping and functional NK cell experiments. **A** EDTA whole blood was stained for cell surface markers and gated initially on FSC/SSC to determine the lymphocyte population. NKG2D expression was subsequently assessed on CD3^-^ CD56^+^ NK cells (both CD56^bright^ and CD56^dim^), CD3^+^ T cells, and CD3^+^ CD56^+^ NKT cells in the patient, the patient’s mother and a healthy control. NKG2D expression on NK cells, CD56^bright^ NK cells, CD3^+^ T cells and CD3^+^ CD56^+^ NKT cells was assessed. **B** The absolute NK cell number was assessed using 6 colour antibody staining and flow cytometry using whole blood. Results were obtained using a FACSCanto II flow cytometer and BD clinical software.

The expression of NKG2D, an NK cell receptor, was assessed on lymphocyte populations; peripheral blood was stained with fluorescently labelled antibodies against CD3, CD56 and NKG2D. NKG2D expression was assessed on CD3^-^CD56^+^ NK cell, CD3^+^CD56^−^ T cells as well as CD3^+^CD56^+^ NKT cells. Despite normal NK cell counts, the proband had absent NKG2D expression on NK cells, T cells and NKT cells (Fig. 2A). The proband’s carrier mother was observed to have normal NKG2D expression.

**Fig. 3:**
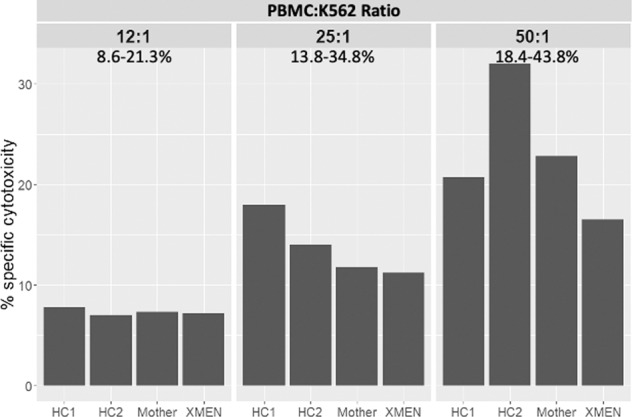
NK killing of K562 cells was intact in the patient and comparable to healthy controls at all PBMC:K562 ratios. PBMC were isolated from whole heparin blood and included in an NK assay against labelled K56s target cell line at three effector:target ratios (50:1, 25:1 and 12.5:1). DNA dye was added at the end of the assay to assess cell death.

As NKG2D is an important receptor expressed on NK cells, we sought to assess NK cell activity. Isolated PBMCs were cultured with labelled K562 cells; after 4 h DNA was stained to determine cell viability. Although lower than comparable samples obtained from healthy controls, cytotoxic activity was close to the normal range at all PBMC:K562 ratios tested (Fig. 3).

## Discussion

In an effort to curtail the “diagnostic odyssey” faced by many rare disease patients, mainstream clinical modalities have embraced the widespread availability of molecular genetic testing. This enabled our identification of a novel *MAGT1* mutation as the underlying cause of XMEN syndrome in a teenage male. Our strategy to create a “virtual gene panel” from the whole-exome sequencing dataset (which comprises a much larger “genomic footprint”), enabled us to flexibly interrogate the increasing number of PID-associated genes. Nevertheless, we concede that facile variant filtering will not always yield the desired diagnostic outcome, and in a recently reported whole-genome sequencing study of more than 1300 PID patients the overall yield was 10.3% [12]. Despite these endeavours we anticipate that the long-term accumulation of diagnostic-grade sequencing datasets, combined with well-curated longitudinal phenotypes, will provide significant further scope for novel disease gene identification. This is consistent with a recent report suggesting many genomic insights remain to be found [13].

The main immunological consequences of this novel mutation have been described and were generally comparable with expected values for T, B and NK populations; they further include our observation of the characteristic inverted CD4:CD8 ratio. In this individual, the T-cell compartment was essentially complete, with no suggestion of CD3^+^CD4^−^CD8^−^TCRαβ^+^ double-negative T cells, as has been described previously described in XMEN patients [5]. Consistent with previous findings, recent thymic emigrants were measured at expected numbers, suggesting normal T-cell development. In this regard, T-cell proliferation was similar to healthy controls, suggesting that T cells are responsive when stimulated with mitogens or via T-cell receptors.

It was anticipated that the anti-CD20 Rituximab treatment, undertaken for lymphoproliferation, would have depleted peripheral blood B cells. Although this was not reflected in our data, it is possible that the B cells were reduced and subsequently repopulated during a gap in our testing, perhaps explaining the expanded transitional B cells (CD19^+^CD24^hi^ CD38^hi^); a characteristic of B-cell populations post chemotherapy. Despite normal age-adjusted B-cell numbers having been observed, the majority were naive, with the memory B-cell compartment reduced beyond what would be expected from a patient approximately 5 years post Rituximab. Whether this is a primary B-cell defect, or secondary to chemotherapy, remains uncertain. It has been previously reported that chemotherapy for some cancers can result in reduced memory B cells frequency due to B-cell depletion and repopulation. The elevated naive and decreased memory B-cell numbers have been reported in other XMEN patients but the reasons remain unclear [14, 15]. It may reflect defective glycosylation of costimulatory T-cell molecules (e.g., CD28) and their reduced cell surface expression which may have downstream consequences on B cells [5]. The patient remained positive for EBV despite receiving rituximab. Although, complete B-cell depletion might follow rituximab treatment, T-cell can continue to be the source of EBV, however, we did not specifically check for this.

NKG2D is a receptor normally found on all NK cells, and a proportion of CD8^+^ T cells. Consistent with previous reports on the immunological status of XMEN patients, we found that both NK cells (CD56^bight^ and CD56^dim^) and T cells did not express NKG2D. CD3^+^CD56^+^ NKT cells were also NKG2D negative. The patient does appear to have a reduced NKT cell population when compared to the healthy control and the mother. However, the percentages of this population are within the age-adjusted normal range, albeit on the lower end of the range [16]. To assess the effect of the absent NKG2D on NK cells, we assayed cytotoxic NK cell activity, using a whole PBMC population using K562 target cells. In contrast to previously reported findings that show significantly decreased NK cell killing, our results suggest that NK killing remains largely intact [6]. However, given the specifics of testing, the results are not directly comparable. In their study, Chaigne-Delalande et al. expanded patient NK cells in vitro with the addition of IL-2 and demonstrated these cells have defective cytotoxicity against K562 cells. The authors further investigated ability to restore NK cytotoxicity with MgSO_4_. NK cells were cultured with and without supplemented MgSO_4_, in the presence of IL-2, for 5 days. It was demonstrated cells cultured with MgSO_4_ media had restored NK killing, however, the NK cells cultured in the absence of MgSO_4_ had a reduction of almost half in % specific lysis of K562 cells when compared to healthy controls. These findings highlighted a significant reduction in cytotoxicity of cultured NK cells in the presence of IL-2. We did not test the NK activity following IL-2 NK activation or CD28 T-cell activation and consequently tested spontaneous NK killing as opposed to NK killing following NK activation.

An inability to clear EBV, and subsequent lymphoma development, is a defining characteristic of XMEN syndrome. EBV, one of the most common human viruses, persists for a lifetime without causing significant problems in immunocompetent individuals. Control of EBV requires a fine balance between the immune system, and viral immune evasion, with NK cells playing a crucial role. NK cells expand following primary EBV infection with killing of infected cells being associated with a downregulation of MHC class I and upregulation of NKG2D ligand (ULBP-1) and other costimulatory molecules (e.g., CD70) on infected cells in the viral lytic stage and the resulting shift in the NK cell signals from inactive to active [17].

NKG2D is one of many NK cell surface receptors, which either initiate an ‘activate’ or ‘inhibit’ signal upon ligand binding. NK cells activation is a consequence of signals from a large repertoire of cell surface molecules. Although NKG2D plays a crucial role in the killing of EBV-infected cells, other signalling molecules may also be involved in generating an overall ‘activate’ signal. Our results suggest that in this patient, signals from receptors other than NKG2D were sufficient for K562 specific cytotoxicity. It remains unclear whether cytotoxic NK activity, against more relevant target cells, would be compromised for our patient. Previous studies have shown that XMEN EBV-specific cytotoxic T lymphocytes had impaired killing of autologous lymphoblastoid cell lines, following expansion via serial stimulations with irradiated autologous lymphoblastoid cells [6].

It has been previously shown that NKG2D alone was not sufficient to initiate the activation of NK cells, requiring a secondary signal [18]. Ravell et al showed a decrease in CD70, CD28, and TCR glycosylation in T cells of XMEN patients, perhaps indicating that in addition to the decrease in NKG2D, the loss of glycosylation of other signalling molecules may be important to the increase susceptibility to EBV in XMEN patients. This is highlighted in patients with CD70 mutation who have an increased susceptibility to EBV-associated lymphomas [19]. While CD70 is expressed at relatively low levels in healthy B cells, EBV infection induces its upregulation and consequently targets the cells for T cells cytotoxicity via TCR-CD27-dependent co-stimulation [20]. A downregulation or impairment in CD70 glycosylation on EBV-infected B cells may hinder the interaction with CD27 on T cells and the subsequent activating signal. NK cells also express CD27 and may similarly have impaired cytotoxicity in response to defective CD70 glycosylation [21].

We assessed lymphocytes from the patient’s carrier mother, using the same range of tests; all analyses were normal. This included all T and B-cell phenotyping, as well and NKG2D expression, and NK cell activity. These results were expected and are concordant with previous reports; lyonization (the random silencing of a single X chromosome in females) provides selection against cells in which the mutation-containing allele is active, resulting in skewed X chromosome inactivation.

In summary, we advocate detailed molecular genetic investigations be performed for all cases of suspected PID. This will require continued investment in our specialty, and an increasingly close working relationship with pathologists, whose expertise sits outside our standard clinical practice. This case also demonstrates the value of performing specialist immunological assays to in patients with novel mutations, to both chart the natural history of the condition and help assess the efficacy of patient treatments.

## Supplementary information

Figure legends

Suppl_Figure 1

Suppl_Figure 2

## Data Availability

The authors confirm that the data supporting the findings of this study are available within the article and its Supplementary material. Raw data that supports the findings of this study are available from the corresponding author, upon reasonable request.
