# Supplementary material for: Identification of a novel MAGT1 mutation supports a diagnosis of XMEN disease
Source: Genes Immun. 2022 Mar 9;23(2):66–72. doi: 10.1038/s41435-022-00166-8 (PMC9042700; doi:10.1038/s41435-022-00166-8)
Supplement: Supplementary file 1 — Figure legends [file 41435_2022_166_MOESM1_ESM.docx]

**Supplementary Figure 1: (A)** Lymphocyte subsets were assessed using 6 colour T/B/NK antibodies and Trucount tubes (BD) using BD clinical software and a FACSCanto II flow cytometer. **(B)** Proliferation was assessed by 3H thymidine uptake of PBMCs following culture in the presence of PHA or anti-CD3.

**Supplementary Figure 2: (A)** Patient’s B cell numbers were assessed following antibody staining of whole EDTA blood and flow cytometry (BD 6 colour TBNK antibody mix and Trucount tubes) using BD clinical software. **(B)** Immuoglobulin subclass levels were measured by nephelometry (BN II, Siemens Helthineers). **(C)** Whole blood was incubated with antibodies to identify B cell subsets (CD19, CD27 and IgD-see materials and methods). Following a red cell lysis step and washing, cells were assessed for naïve and memory B cell populations by flow cytometry using DIVA. Using these antibodies B cell populations were identified as highlighted in the annotation on the plots.
