# Supplementary figures and images for: Identification of a novel MAGT1 mutation supports a diagnosis of XMEN disease
Source: Genes Immun. 2022 Mar 9;23(2):66–72. doi: 10.1038/s41435-022-00166-8 (PMC9042700; doi:10.1038/s41435-022-00166-8)

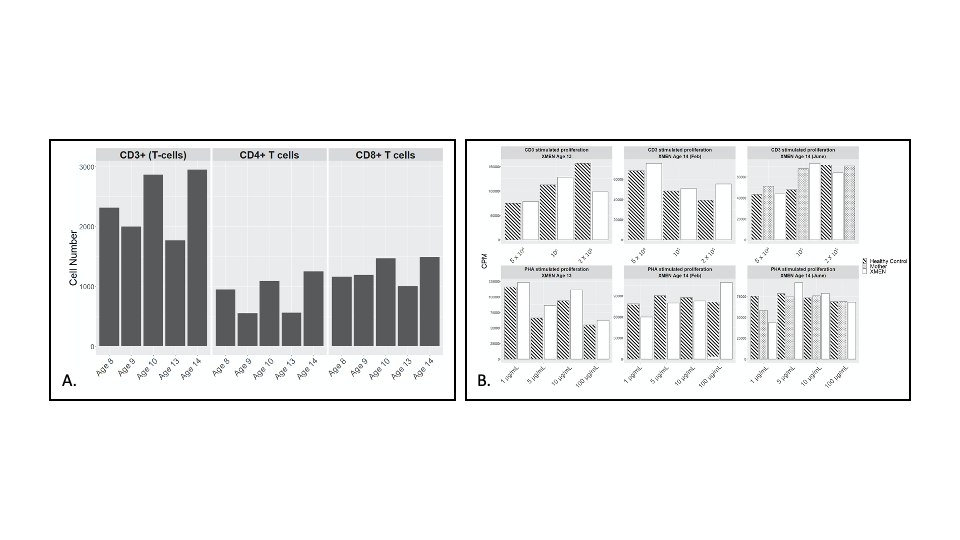

Supplement: Supplementary file 2 — Suppl_Figure 1 [file 41435_2022_166_MOESM2_ESM.tif]

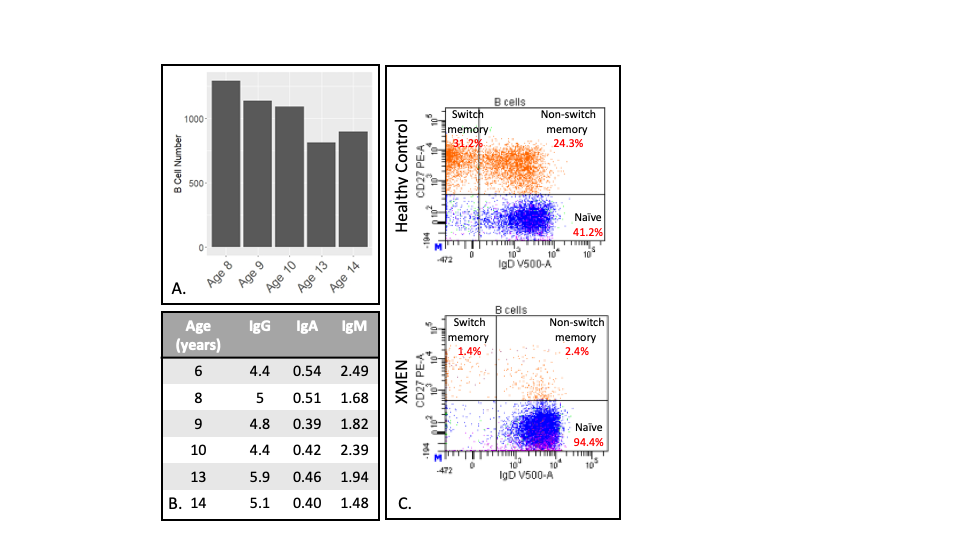

Supplement: Supplementary file 3 — Suppl_Figure 2 [file 41435_2022_166_MOESM3_ESM.tif]
